# On the Interaction of the Photovoltaic Response With Ferroelectric and Magnetic Domains: Magnetoelectric Control of the Photovoltaic Response in BiFeO_3_ Thin Films

**DOI:** 10.1002/smtd.70625

**Published:** 2026-03-24

**Authors:** Luciano Cardoso Dias, Eduardo Astrath Volnistem, Maurício Mazur, Fernanda Barbieri, Karina Midori Endo, Ana Carolina Ferreira, Mariele Noemi da Silva Diaz, Taiana Gabriela Moretti Bonadio, Ricardo Yoshimitsu Miyahara, Tania Toyomi Tominaga, Gustavo Sanguino Dias, Luiz Fernando Cótica, Ivair Aparecido dos Santos, José Antonio Eiras, Valdirlei Fernandes Freitas

**Affiliations:** ^1^ Graduate program in Physics Federal University of Parana Curitiba PR Brazil; ^2^ Graduate Program in Physics State University of Maringá Maringá PR Brazil; ^3^ Graduate Program in Nanoscience and Biosciences Midwestern Paraná State University Guarapuava PR Brazil; ^4^ Graduate Program in Physics Federal University of São Carlos São Carlos SP Brazil

**Keywords:** bismuth ferrite, ferroelectric domains, magnetoelectric coupling, photovoltaics, thin Films

## Abstract

In the search for innovative photovoltaic solutions, bismuth ferrite (BiFeO_3_) has received significant attention due to the possibility of simultaneous use of its photovoltaic (PV), ferroelectric, and magnetic properties. Furthermore, the possibility of controlling photovoltaic responses through the orientation of ferroelectric and magnetic domains by external fields gives great potential to devices made up of BiFeO_3_ thin films. This paper investigates PV responses of BiFeO_3_‐based devices under the influence of the different ferroelectric and magnetic domain orientations. We have found a strong correlation polarization/magnetization states with PV. This relation gives rise to a huge increment in photogeneration that goes up to about 7‐fold higher than in pristine samples. Thus, BiFeO_3_‐based photovoltaic devices can be highlighted as tunable, multifunctional, and amplifiable systems by electric and magnetic stimuli.

## Graphical abstract

This study demonstrates tunable photovoltaic responses in pure, polycrystalline BiFeO_3_ thin films, controlled by external electric and magnetic fields. By aligning ferroelectric domains and leveraging magnetoelectric coupling, the photogeneration is amplified up to seven times. These findings highlight the potential of cost‐effective, solution‐processed multifunctional devices with non‐volatile magnetic and electric control systems, promising for sustainable energy applications.

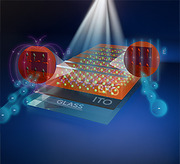

## 1 Introduction

In recent years, a pressing need for innovative energy solutions has arisen due to the increased release of environmentally harmful gases, unbridled exploitation of natural resources, and heightened energy consumption driven by technological advances [1, 2]. This has prompted a heightened focus on clean and renewable energy sources, with solar cells emerging as a beacon of hope among these sustainable technologies [2, 3]. However, some conventional solid‐state photovoltaic (PV) materials are unable to produce voltages higher than their electronic bandgap [4]. In this context, oxide ferroelectrics with a perovskite structure (ABO_3_ type) have gained significant attention in the field of PV applications over the last few years [5, 6], due to the ability of specific ferroelectrics of generating PV responses that surpass the intrinsic material band gap (anomalous effect), a unique feature associated with the ferroelectric photovoltaic (as called bulk photovoltaic effect—BPV) effect [4, 7]. This effect is generated by the internal electric fields of a non‐centrosymmetric polarized state of ferroelectric material, which can readily separate photogenerated electrons and holes. These positive and negative charges create an electric potential difference that may be even greater than the bandgap of the material itself, leading to higher open‐circuit voltages (V_OC_). Moreover, the PV effect in ferroelectric materials does not depend on the existence of a conventional p‐n junction, thereby escaping from the imposed limits for the conventional PV response [4, 8, 9, 10]. In fact, the potential of these BPV responses holds the key to transforming the next generation of PV devices, potentially overcoming the limitations outlined by the Shockley–Queisser limit in conventional p‐n junction‐dependent devices [9, 11].

Among the oxide ferroelectrics with perovskite structure, bismuth ferrite (BiFeO_3_, hereinafter BFO) stands out as a notable choice to constitute multifunctional PV‐based devices [5, 8]. In fact, BFO shows ferroelectric and magnetic properties along with a strong magnetoelectric coupling that can be used individually or together to improve and/or control the PV response. This singular PV feature arises from the BFO non‐centrosymmetric crystal structure (*R*3*c* space group), which results in a spontaneous ferroelectric polarization with a high‐temperature ferroelectric phase transition occurring at T_C_ = 830°C, typically resulting in a high saturation ferroelectric polarization, reaching ∼100 µC/cm^2^ in BFO thin films [12, 13, 14, 15]. This unique characteristic is a consequence of the interaction between the Bi 6s^2^ and the O 2p orbitals, leading to the displacement of the center of symmetry of positive and negative charges at the BFO rhombohedral (*R*3*c*) unit cell [14, 15]. Furthermore, BFO shows an antiferromagnetic ordering below T_N_ = 370°C. This combination of ferroelectric and antiferromagnetic states renders to BFO a remarkable multiferroic state that remains active at room temperature [14, 15, 16]. Moreover, it is widely recognized that BFO can also exhibit a weak ferromagnetic state, which is the result of small inclinations in the magnetic moments within its G‐type antiferromagnetic backbone [16, 17]. In addition, a magnetoelectric coupling is also observed in BFO in several configurations, including doped bulk [17, 18, 19, 20], thin films on epitaxial substrates [21], and BFO‐doped thin films [22, 23, 24]. It is worth mentioning that the use of the BFO magnetoelectric coupling can be a path to be explored for enhancing the generation of the PV effect in BFO‐based devices. Finally, BFO is known to show 71°, 109°, and 180° domain walls (DW). The estimated wall energies follow the order: 109° < 180° < 71°, indicating that domain reorientation involving a 109° DW is energetically more favorable [25].

Numerous studies have been reported in the existing literature which demonstrate the enhancement of the PV response and reduction of leakage current in BFO by various methods, including doping [26, 27, 28], bandgap engineering [29], absorption control [30], domain engineering [31, 32, 33], and multilayered films [34, 35]. Moreover, ferroelectric polarization plays a crucial role in enhancing the PV response, mainly due to the rearrangement of electrical charges that act as a PV amplifier, creating electrically favorable regions that reduce the recombination of electron‐hole pairs [25, 36].

Nonetheless, it is still crucial to determine the basic photovoltaic behavior of pure, polycrystalline thin films under domain orientation. The potential of multiferroic coupling (using both electric and magnetic fields) is still an unexplored path for enhancement, even though several studies have used external electric fields (poling) to manipulate PV responses [25, 34, 37, 38]. To demonstrate that magnetoelectric control of the PV response is achievable even in simple solution‐processed films, this work provides a robust baseline for future multifunctional systems.

We investigated the photovoltaic response in BFO‐based thin‐films devices with the orientation of ferroelectric domains induced by external electric and magnetic fields. Impressive responses were observed through light‐illuminated scanning probe microscopy and conventional photoresponse measurements in both magnetized and electrically poled devices. Under illumination in a Kelvin probe force microscope (KPFM), samples whose polarization inversion was promoted, confirmed by piezoelectric force microscopy (PFM), revealed an increase in photogeneration of up to 7 times. In addition, when magnetized, antiparallel PV responses indicated strong magnetoelectric coupling that locks the polarization directions (electric dipoles) perpendicular to those of magnetization (magnetic dipoles). The mechanisms relating the photovoltaic responses to the orientation of domain walls and domains are compared with PN junctions’ behavior and carefully discussed. These novel outcomes advance BFO‐based PV devices as prospective multifunctional devices that are smart, adjustable, enhanced, and in which the PV response might be controlled by external electrical or magnetic stimuli.

## 2 Results and Discussion

**FIGURE 1:**
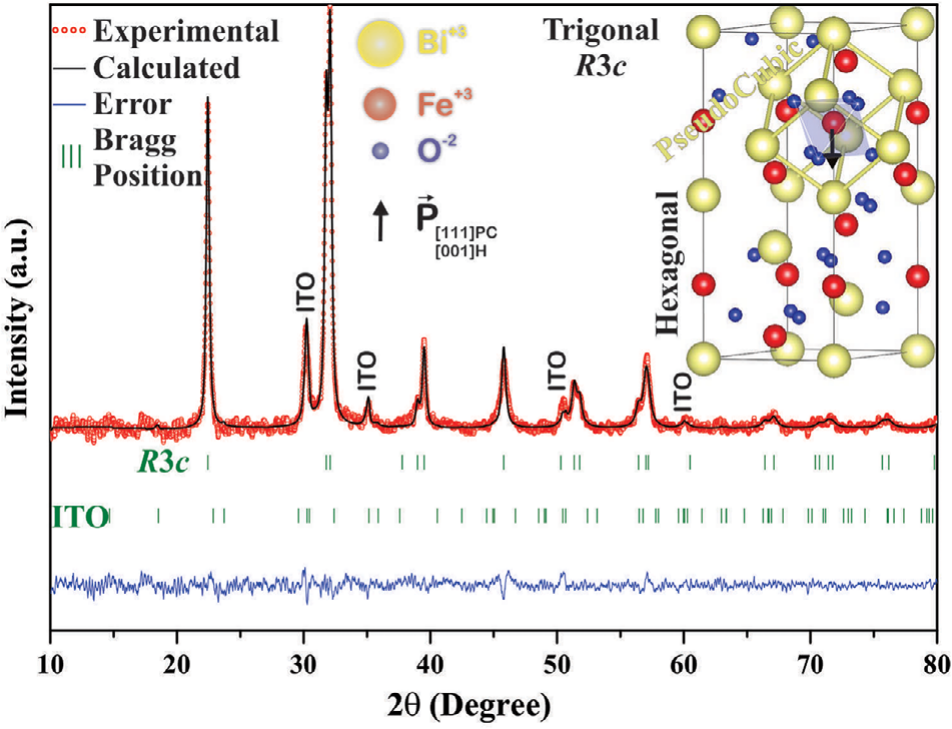
X‐ray diffraction pattern and Rietveld refinement results for the BFO/ITO/Glass device. Inset: Trigonal crystal (*R*3*c* space group) highlighting the pseudocubic and hexagonal symmetries.

Figure 1 shows the X‐ray diffraction (XRD) data and the Rietveld refinement results (experimental and calculated patterns, errors, and Bragg positions) for the BFO thin film deposited on a tin‐doped indium oxide ([In_1.88_Sn_0.12_]O_3_ – ITO)/glass substrate (BFO/ITO/Glass configuration). The data was smoothed, and the amorphous background, referring to the glass substrate, was removed from the experimental result to improve the visualization of the BFO crystalline peaks. The original data is presented in supplementary material S1. As expected, the observed diffraction pattern is composed by the crystalline pattern of the BFO and ITO thin films, superimposed on the amorphous pattern of the glass substrate. The diffraction pattern was only obtained in the pristine state of the BFO film, that is, before the electric or magnetic field applications. In this state, no significant preferred orientation was observed in the diffraction pattern. The experimental pattern was best fitted using a trigonal crystal structure (*R*3*c* space group) for the BFO thin film, along a cubic symmetry (*Ia*‐3) for the ITO. The inset of Figure 1 illustrates the two main symmetries of the trigonal crystal. It is worth mentioning that trigonal crystals, like the *R*3*c* ones, are understood by three coexisting structural symmetries, hexagonal, rhombohedral, and pseudocubic. Two of them, hexagonal and pseudocubic, are of special interest for this work because the hexagonal one is used in Rietveld refinement due to its several degrees of freedom, while the pseudocubic symmetry, used in Geometrical Polarization Approach (GPA) analysis, is the polar arrangement where the intrinsic polarization can be obtained.

To quantify the spontaneous intrinsic polarization of the BFO thin film deposited on an ITO/Glass substrate, the GPA method [39] was applied using the structural parameters obtained from the Rietveld refined XRD data. The results are summarized in Table 1, which also lists the spontaneous polarization determined via GPA method. A high spontaneous polarization of ∼ 58 µC/cm^2^ was observed, being in good agreement with recent reports for polycrystalline films synthesized under similar conditions. For instance, Yi et al. [40] reported values around 65 µC/cm^2^ for films processed without oxygen atmosphere control. Our result confirms that the GPA method effectively estimates the intrinsic polarization expected for solution‐processed BFO films.

**TABLE 1:** Rietveld refinement results for the BiFeO_3_ compound deposited on the ITO/Glass substrate. From top to bottom: Structural parameters: Space group, lattice parameters for the hexagonal (*a_H_*, *b_H_* and *c_H_*) and pseudocubic (*a_PC_*) symmetries, atomic positions (X, Y, and Z) of hexagonal symmetry, and Rietveld reliability factors (R_Bragg_, R_F,_ and χ^2^). GPA parameters: Effective electric charge (*q_eff_*), cation and anion average positions (*Pos_CAT_ and Pos_ANI_*), and, lastly, polarization vector (*Vet_POL_*) projected in hexagonal and in pseudocubic symmetries. Finally, intrinsic polarization for the pseudocubic unit cell.

Structural Rietveld			
Parameters	Values		
Space Group	*R*3*c*		
*a*_H_ [Å]	5. 5769(5)		
*b*_H_ [Å	5. 5769(5)		
*c*_H_ [Å]	13.8568(6)		
*a*_PC_ [Å]	3.9579(9)		
Atomic Positions			
	X	Y	Z
Bi	0.0000	0.0000	−0.0144
Fe	0.0000	0.0000	+ 0.2063
O	+0.5953	+ 0.0828	− 0.9437
Reliability factors			
R_Bragg_	5.87		
R_F_	4.56		
χ^2^	1.16		
Geometric Polarization Approach			
***q_eff_* [10^−19^ C]**	9.61(3)		
***Pos_CAT_* [10^−10^m]^Hexagonal^**	5.570950 x̂ + 5.570950 ŷ + 10.06762 ẑ		
***Pos_ANI_* [10^−10^ m]^Hexagonal^**	5.570950 x̂ + 5.570950 ŷ + 9.687860 ẑ		
***Vet_POL_* [10^−10^ m]^Hexagonal^**	0.000000 x̂ + 0.000000 ŷ + 0.379760 ẑ		
***Vet_POL_* [10^−1^m]^Pseudocubic^**	0.219255 x̂ + 0.219255 ŷ + 0.219255 ẑ		
***P* [*μ.C/cm^2^*]^Pseudocubic^**	**58.87**		

The vectors defining anion positions (***Pos_ANI_***), cation position (***Pos_CAT_***), and polar vector (***Vet_POL_***), are described relative to hexagonal symmetry such that the dipole moment aligns along the [001]H direction. With some vector algebra [41], one can derive the equivalent polarization vector in pseudocubic structure that would reveal spontaneous polarization to be aligned along the [111]_PC_ direction. Polarization information is collected directly from the polar pseudocubic symmetry, which, in this protocol, is described/located by using the parameters of the hexagonal symmetry of the trigonal crystal (inset – Figure 1). In fact, it is possible to describe the internal pseudocubic symmetry using specific equivalent positions, within the symmetry operations, from the refined structural parameters for hexagonal symmetry (see ref [42] for detailed discussion). Therefore, the lattice parameters listed in Table 1 refer to those obtained from hexagonal symmetry (*a*, *b*, and *c*) as well as the atomic positions for Bi, Fe, and O (X, Y, and Z). Finally, the Rietveld refinement reliability factors confirm an excellent agreement between the experimental and calculated patterns, as evident in the graphical criterion (blue line) shown in Figure 1.

**FIGURE 2:**
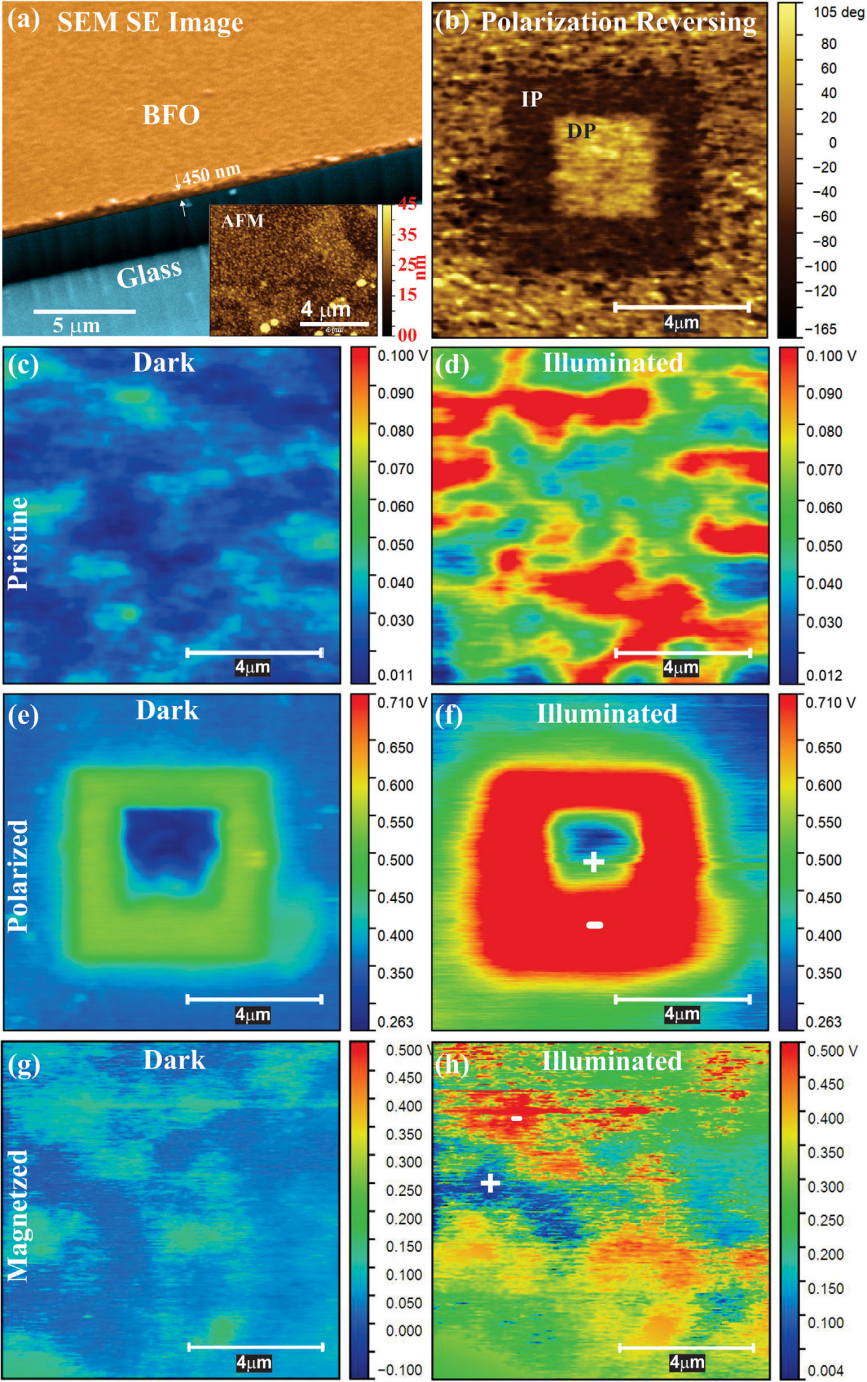
Scanning probe microscopies for the BiFeO_3_ thin film on the ITO/Glass substrate. (a) SEM and AFM images, (b) directly and inversely poled squares in the PFM analysis, (c) KPFM image of the *dark* pristine surface, (d) KPFM image of the *illuminated* pristine surface, (e) KPFM image of the *dark* surface on the square of poled area, (f) KPFM image of the *illuminated* surface on the square of poled area, (g) KPFM image of the *dark* surface on the directly magnetized area in the remanent state, and (h) KPFM image of the *illuminated* surface on the directly magnetized area in the remanent state.

**FIGURE 3:**
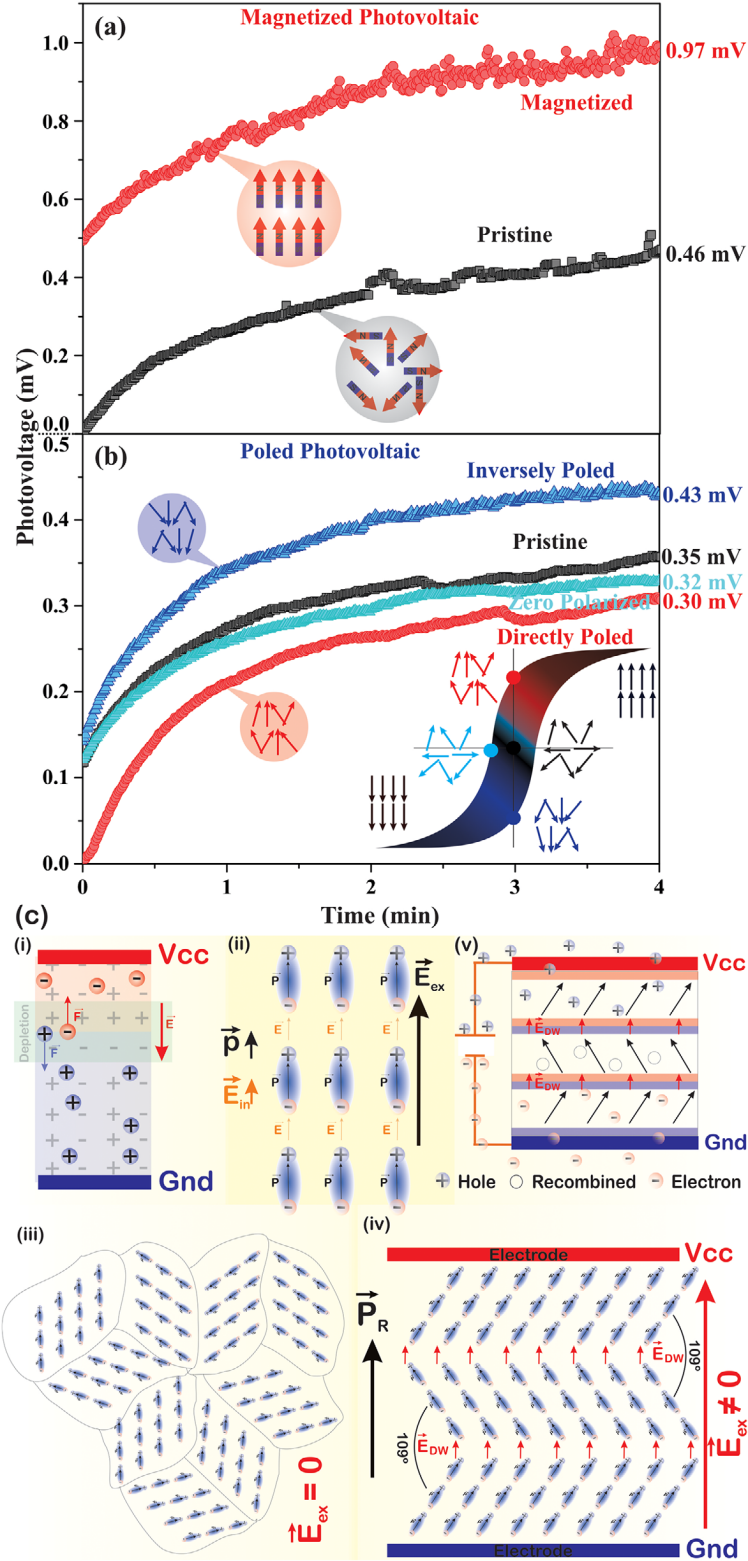
Time dependent photovoltage for BFO/ITO/Glass devices under different conditions: (a) applying an external magnetic field (magnetized photovoltaic). The insets illustrate the corresponding magnetic dipole alignments for the pristine (random)and magnetized (aligned) states. (b) applying an external electric bias field (poled photovoltaic). The insets show a schematic ferroelectric hysteresis loop illustrating the corresponding local domain orientation states for each poling condition. (c) Schematic representations of the photovoltaic response mechanisms: (i) conventional PN Junction charge separation, (ii) locally oriented ferroelectric dipoles, (iii) random domain wall arrangement without an external field (E_ex_ = 0), (iv) parallel orientation of domain walls with electrodes under an applied external field (E_ex_ ≠ 0) and (v) photovoltaic mechanism in ferroelectric materials.

Scanning probe microscopy (SPM) analyses were carried out to observe the effect of the poling process on the PV response of the BFO/ITO/Glass device, and the results are presented in Figure 2. A surface secondary electrons (SE) image was obtained from Scanning Electron Microscopy (SEM) analysis in the BFO/ITO/Glass device, and a thin film of BFO (thickness around 450 nm) was observed, as shown in Figure 2a. In addition, nanometric grains (∼ 150 nm) were also identified in the Atomic Force Microscopy (AFM) image (Figure 3a, inset (i)), indicating that the BFO thin film is polycrystalline.

Ferroelectric domains with directly (DP) and inversely poled (IP) regions (Figure 2b) were analyzed by PFM within a square region of approximately 3 × 3 µm, and 6 × 6 µm, respectively. First, an external bias of + 1 V (∼ 2.2 kV/mm) was first applied to orient the ferroelectric dipoles/domains in the downward direction (IP), as seen by the black square in Figure 2b. To reverse the dipole/domains orientation (DP), a ‐1 V bias was applied in the central region of the previously poled square, resulting in upward‐oriented dipoles/domains (3 × 3 µm yellow square in center of Figure 2b). The highlighted contrast between these two poled regions demonstrates that a well‐defined reversal of dipoles under an external electric field is obtained, thereby confirming the ferroelectric nature of BFO thin film.

KPFM has been used to detect the surface potential of BFO thin film under dark and illuminated conditions for pristine, polarized, and magnetized devices. In the pristine state (Figure 2c), the film exhibited surface potential values ranging from 0.011 to 0.040 V under dark conditions. Upon illumination (Figure 2d), this range increased to 0.012 V – 0.100 V, which is related to the PV effect in BFO. Analyzing the polarized state (Figure 2e), a clear organization of electric dipoles was observed, in agreement with PFM measurements. A significant increase in the surface potential was observed, reaching approximately 0.500 V in IP regions (green) and 0.263 V in DP regions (blue). The bigger potential (green region) is a consequence of the alignment of the electric charges that form the electric dipole in the polarization process. Furthermore, under illumination (Figure 2f), a remarkable enhancement of around 1.5 times is recorded in the IP region, where potential jumps from ∼ 0.500 to 0.700 V. This value is approximately seven times higher than that of the illuminated pristine surface (Figure 2d). Such behavior indicates that the polarization effectively modulates the electrical response of the BFO thin film, directly influencing its photovoltaic properties.

Another interesting feature of the poling process and its influence in PV responses is the homogeneity of the device's surface response when fully polarized. Specifically, when the entire surface is electrically poled, it exhibits a uniform PV response across the entire area. This is evidenced by the nearly constant surface potential observed in the polarized regions under illumination (red regions in Figure 2f), which differs from the inhomogeneity of the non‐polarized surfaces (scattered red regions over the surface in Figure 2d). Finally, all these phenomena attest to the multifunctional character of the BFO‐based PV device, which acts as a PV‐FE, strongly influenced by the microscopic ferroelectric polarization of the BFO active layer.

Conversely, to study the impact of direct magnetization, a north pole magnetic field (direct magnetization) was applied and subsequently removed prior to KPFM characterization under dark/illuminated conditions, as shown in Figure 2g,h, respectively. Notably, in comparison with the pristine device (Figure 2d), under illumination, the magnetized film presented a surface potential of around 0.500 V (Figure 2h—red regions) in specific regions, that is five times bigger than the dark one (Figure 2g – 0.100 V in light blue regions). This behavior contrasts with that observed in the electrically poled state, where the entire polarized square exhibited a uniform PV response (Figure 2f). Furthermore, not all magnetized regions shown a significant increased surface potential (Figure 2h). This can be associated with the low magnetic remanence of BFO, which typically exhibits a weak/antiferromagnetic hysteresis loop at room temperature [43]. Consequently, while direct electrical poling yields a highly uniform and intense KPFM contrast due to BFO's strong ferroelectricity, the magnetoelectric coupling mediated by a magnetic field produces a weaker and more heterogeneous surface potential response, reflecting the material's low magnetic remanence.

These results suggest that the surface charge accumulation of electric in the PV device is controlled by the orientation of electric dipoles and ferroelectric domains within the BFO thin film. This orientation can be externally tuned by applying electric and/or magnetic fields. This behavior offers additional evidence of the magnetoelectric coupling in BFO, wherein magnetic ordering can influence local electric polarization and consequently, the PV response.

The macroscopic PV responses of the Hg/BFO/ITO/Glass device in pristine, electric poled, and magnetized conditions were analyzed, and the results are shown in Figure 3. The photovoltage of the magnetized device reveals a significant increase compared to the pristine one (Figure 3a). Interestingly, the photovoltage under illumination starts at 0.5 mV for the magnetized device.

This suggests that the remaining ordering of the magnetic dipoles in BFO may be responsible for influencing the alignment of the electric dipoles, promoting photovoltage in short times (0.5 mV in this case). In fact, as observed by KPFM analyses, some electric dipoles/domains are rearranged parallel to z‐direction, resulting in this 0.5 mV background. Remarkably, these effects are characteristic of magnetoelectric coupling, in which the alignment of electric dipole moments is connected to the magnetic dipoles by 90° [44].

The Hg/BFO/ITO/Glass device was subjected to different electrical poling processes, and its time‐dependent photovoltage responses were subsequently analyzed, as shown in Figure 3b. In this arrangement, the PV responses in the DP and IP device were evaluated to schematically represent the ferroelectric hysteresis loop protocol (inset, Figure 3b), orienting the ferroelectric domains of the BFO layer, such as in a hysteresis cycle. Thus, the photovoltage was first determined in its pristine state (black curve), followed by IP state (application of an electric field of + 0.35 kV/mm for 30 min, blue curve). Following that, the first step DP (zero polarized) state was also analyzed (application of an electric field of – 0.35 kV/mm for 30 min, light blue curve), ending with DP state (application of an electric field of – 0.35 kV/mm for more 30 min, red curve) as shown in Figure 3b.

It is important to note that a small variation in the pristine state photovoltage is observed between the measurements shown in Figure 3a,b. This variation is attributed to some local strain conditions, defect distribution, and minor structural differences inherent to the polycrystalline nature of the solution‐processed films. However, despite these slight initial differences, the focus here is on the relative modulation of the photovoltaic response under external stimuli, which demonstrates the functional tunability of the devices.

Looking at the energetic aspects of the obtained curves, Figure 3b might suggest that the applied energy may, at first glance, seem low. However, domain walls within ferroelectric materials are anchored with different energies, as they occur under different conditions, such as defects, vacancies, dopants, contaminants, interdomain connection structures, particle sizes, and synthesis processes, among others. All these conditions are examples of what can affect the energy required to unanchor domain walls when oriented by external fields. Furthermore, new low‐energy regions or different energies are also induced by external stimuli. Therefore, even if the hysteresis curve indicates a certain anchoring electric field strength, such as the coercive field or its saturation, lower energies also directly affect the dynamics of ferroelectric domain walls.

Going back to analyze the curves, the IP state exhibits the highest PV response, exceeding both pristine and DP states. This is attributed to the alignment of electric dipoles/domains perpendicular to the device surface, consistent with previous scanning probe microscopy analyses (Figure 2f,h). Furthermore, after reversing the electric field in the first step DP state, the photovoltaic response returns to values similar to the pristine state. This suggests that the applied field intensity and duration were insufficient to induce a fully stable DP alignment or to fully erase the IP state by the application of the inverse electric field. However, after an additional 30 min DP polarization process, the lowest photovoltage response was achieved (Figure 3b, red curve), indicating the successful formation of the DP state. It is important to note that “none” electric field was applied during the measurement of the photovoltaic responses, confirming that the observed changes are intrinsic to the material's ferroelectric polarization state.

The mechanisms proposed to describe the photovoltage responses in polarized ferroelectrics, as in our Hg/BFO/ITO/Glass device, are illustrated in Figure 3c. The forces acting on this system, generating useful photovoltaic response, come from intrinsic internal electric fields formed by the orientation of internal electric charges, the bulk effect. This arrangement is similar to that observed in the PN junctions in conventional silicon solar cells. However, here the arrangement of charges that generate a built‐in electric field originates from the ferroelectric polarization of the BFO. To better understand, the well‐known photovoltaic mechanisms for PN junctions are illustrated in Figure 3c, inset (i). The built‐in electric field in the depletion zone separates electron‐hole pairs that are created by light in this junction. Electrons move toward the N‐side, and holes move toward the P‐side. This creates a potential barrier that prevents recombination. This charge separation makes it possible to get electrical energy, which powers an electrical circuit. This is known as the “useful photovoltaic effect”.

In ferroelectric materials, an analogous process occurs, where the internal electric field and domain walls act like a depletion zone, to form an intrinsic electric field that prevents the electron‐hole recombination. When a ferroelectric material, such as BFO, is synthesized, it spontaneously forms ferroelectric domain regions (Figure 3c, inset (ii)—ordered ferroelectric dipoles) without any external electric field (**E_ex_**) through a process of internal force equilibrium. Between one domain and another, so‐called ferroelectric domain walls are formed, originating from the arrangement of charges in which an electric field emerges, here called **E_DW_**. However, the domains are randomly oriented, as shown in Figure 3c, inset (iii). These **E_DW_** fields generate a potential barrier throughout the polarized volume of the ferroelectric, that prevents electron‐hole recombination, generating the useful PV effect by the so‐called bulk photovoltaic effect (BPV) [25, 26]. Notwithstanding, this process is further complicated by the random orientations of the ferroelectric domains of the material (Figure 3c, inset (iii)). Despite this, when an external electric field is applied, these domains are reoriented in the direction of the **E_ex_** if their internal energy is overcome by the applied external field (Figure 3c, inset (iv)). Upon removal of the field, the remanent polarization (**P_R_**) acquires a vectorially preferred orientation perpendicular to the electrodes due to the action of the internal field forces. Consequently, the domain walls (DW) are positioned parallel to the electrodes, as illustrated in Figure 3c, inset (iv). Notably, in BFO, domain walls preferably form at 109° angles, leading to head‐to‐tail dipole arrangements that generate domain wall fields (**E_DW_**).

In this arrangement, when **P_R_** is oriented perpendicular to the electrodes and the DW are oriented parallel to them (Figure 3c, insets (iv) and (v)) the useful PV is favored [4]. As illustrated in Figure 3c, inset (v), in the head‐to‐tail dipole orientations DW an intense electric force is generated by the action of the **E_DW_**, driving electrons and holes in opposite directions, analogous to charge separation in a PN junction depletion zone. However, in intermediate domain regions, as in multidomain‐arranged samples, i.e., polycrystals, the electrons from the lower DW could become trapped by the **E_in_** (electric fields formed by the charges that form the dipole in the body of the domain) or, more probable, recombine with the holes from the upper DW, resulting in charge neutralization (empty circle in Figure 3c, inset (v)). In contrast, in the upper and lower domain regions, charge carriers remain completely separated, becoming photovoltaic energy useful for powering an electric circuit (useful PV effect), as shown in the Figure 3c, inset (v).

A more detailed examination of the DW configuration in BFO can provide insights into the PV phenomena observed in this study. It is known that the DW in BFO can be oriented at 71°, 109° and 180°, and that their energies (λ) theoretically estimated as λ109° < λ180° < λ71° [25]. This means that 109° DW are easiest to create/orient/move under an **E_ex,_** which makes this the most likely configuration to happen. Earlier research [45] has demonstrated that the PV response (V_OC_) in BFO is significantly influenced by the relative orientation of DW and electrodes. In other words, when 109° DW are parallel to the electrodes, the photovoltaic effect is much stronger, contributing to the useful PV effect. However, when DWs are perpendicular to the electrodes, the photovoltaic response drops drastically, almost to zero.

In this study, instead of positioning the electrodes parallel to the DW, the polarization process itself was responsible for aligning the DWs parallel to the electrodes. Thus, the photovoltaic responses observed in the poled BFO device (Figure 3b) can be understood considering the proposed mechanisms of Figure 3c. When the device is in the direct poled state, the orientation of the domains and DW favors the useful PV effect, leading to the higher PV responses, as shown in Figure 3b. However, when the device is reversely poled, the PV response decreases. In fact, this response does not imply that polarization does not work in the reverse direction, instead, the electric flow reverses its direction, and the electrons go to the highest potential (V_cc_) in the circuit. In this configuration, electrons do not pass through the voltmeter, which in this case acts as the electrical load to be powered. It is probably for this reason that the photovoltage curve in the reversely poled device decreases in amplitude.

Naturally, while the dominant mechanisms described above primarily govern the observed PV responses, other physical mechanisms (pyroelectricity, piezoelectricity, etc.) likely play a role, albeit with varying probability, even in the reversely poled state. In fact, as an example, the remanent polarization does not extend across the entire volume of the ferroelectric material. Instead, only specific regions of the device remain oriented. Furthermore, polarization dynamics encompasses different effects, such as the orientation of regions that were not previously oriented, reorientation of already polarized domains, the movement of other DW types (71° and 180°) occurring with low probability; creation/induction of new low‐energy domains under an **E_ex_**, oriented domains forming uniformly align regions originated from other phenomena, and so on. All these things can happen statistically and affect how ferroelectric‐based PV devices respond to light. The ferroelectric capacitor effect is one last important factor that could explain the responses that were seen. The intrinsic capacitance in ferroelectrics is likely the reason why the PV response slowly gets stronger over time. This mechanism traps photogenerated electron‐hole pairs in the internal fields (**E_in_**) linked to domains and DW, creating small potential differences that increase with extended light exposure. Consequently, the PV response is noted to increase over time, consistent with a capacitive charging phenomenon in ferroelectric‐based photovoltaic devices.

The photovoltage measurements obtained under different magnetic conditions reveal a direct and systematic influence of magnetization on the photovoltaic response of the samples. In this way, the BFO device was also subjected to a magnetization process using a permanent DC magnetic field, and the resulting photovoltaic responses under this condition were analyzed. Although the magnetic ordering induced by the field does not remain fully stable after field removal, relaxing instead toward intermediate states. It is reasonable to assume that the electric dipoles, which become partially aligned through magnetoelectric coupling during magnetization, also undergo a relaxation process. Nevertheless, a certain degree of both magnetic and electric ordering persists even after the external field is removed. This residual magnetic and, consequently, ferroelectric alignment promotes ferroelectric domain orientations parallel to the electrodes and contributes to the amplification of the photovoltaic response observed in the device after the magnetization procedure. In this process, the mechanisms that enhance the photoresponse are expected to be similar to those previously discussed. However, in this case, we believe that they are mediated by the magnetic field through magnetoelectric coupling. Although the modulation of the photovoltaic response by the magnetic field provides clear evidence of the magnetoelectric coupling in the BFO films, a direct quantification of the magnetoelectric coefficient (α_ME_) was not within the scope of this study. Instead, the focus is placed on the functional control and qualitative trends of the photovoltaic response under magnetic stimuli. Regarding the magnitude of this interaction, literature records for polycrystalline BFO films report experimental magnetoelectric coefficients (α_ME_) of approximately 7–10 mV/cm·Oe, while theoretical studies have determined values up to 104 mV/cm·Oe [46, 47, 48].

To summarize, the whole set of results points to a promising device for practical PV applications. The intrinsic polarization, collected via GPA method (Table 1), indicates that the BFO‐based device synthesized in this work is highly polarized (∼ 58 µC/cm^2^). These results are reinforced by the images collected by PFM, which reveal a highly poled surface and the complete reversal of the BFO polarization under the application of an external electric field (poled square of Figure 2b). Such polarization switching is a key signature of the ferroelectric state. Moreover, KPFM analysis under illumination (Figure 2c,d) demonstrated a correlation between the PV responses and the ferroelectric polarization of the BFO film. These results were reinforced by the macroscopic characterization of the photovoltage responses in a poled device (Figure 2b). Moreover, the connection between PV responses and the magnetization of the BFO device (Figure 2e,f and 2a) also indicates an intercorrelation between ferroelectricity and magnetism. In this case, the proposition is that it is an indirect effect, mediated by the magnetoelectric effect, which consecutively guides magnetic and electrical moments under the influence of an external magnetic field. As seen, all these effects are closely connected in the PV device studied. The strong interaction between photovoltaic, magnetic, and ferroelectric properties shows the multifunctional nature of this BFO‐based PV device. In fact, materials whose photovoltage reacts to electric and magnetic fields make it possible to create a new type of hybrid opto‐magnetoelectric technologies. Some important possibilities are “reconfigurable magnetoelectric photovoltaics”, capable of tuning efficiency under external stimuli; “ultrasensitive optical–magnetoelectric sensors” for detecting electric or magnetic fields through light‐induced voltage; and “nonvolatile photovoltaic memories and logic”, where ferroic states are read optically for low‐power neuromorphic architectures. These materials also support “self‐powered devices and spin‐photovoltage detectors”, paving the way for compact, intelligent, and reconfigurable multiferroic systems across energy, sensing, and computing applications.

## 3 Conclusions

The study of BFO‐based photovoltaic (PV) devices showed a strong link between the ferroelectric polarization/magnetic states and the observed PV responses. This led to a significant increase in photogeneration, up to seven times greater than pristine samples. The highest PV amplification is achieved through electric poling, which uses BFO's high intrinsic polarization (∼ 58 µC/cm^2^) to get the most out of the Bulk Photovoltaic Effect (BPV). This poling process successfully aligns the internal domain walls (DWs), especially the energetically favorable 109 ° DWs, parallel to the electrodes. This is a necessary configuration that strongly favors charge separation and the “useful PV effect”. The device's multifunctionality was further evidenced by its response to external magnetic fields, wherein the PV effect is indirectly affected by magnetoelectric coupling that stabilizes electric dipoles perpendicular to magnetic dipoles. The PV response mediated by magnetization was, however, less intense and more heterogeneous than that from electrical poling, due to BFO's low magnetic remanence. It is important to note that all observed PV changes are intrinsic and remanent because no external bias was applied during measurement. This is because the material's internal electric fields are kept in place by the successful domain orientations. These findings support the possibility of using BFO‐based systems as multifunctional, tunable, and amplifiable PV devices that are controlled by electric and magnetic stimuli. This demonstrates the potential of simple synthesis methods to create devices where the PV response is effectively tuned by magnetic domains, without the need for complex substrate engineering.

## 4 Experimental Methods

### 4.1 Solution Synthesis

BiFeO_3_ (BFO) thin films, deposited on ITO‐Glass substrates (Sigma Aldrich – 70–100 Ω/sq), were prepared using a modified Pechini chemical method followed by dip coating, as previously reported [49]. To prepare the BFO solution, bismuth III (Bi(NO_3_)_3_‐5H_2_O – 98.00%) and iron (Fe(NO_3_)_3_‐9H_2_O – 98.00%) nitrates were initially dissolved (1:4 ratio) in citric acid (C_6_H_8_O_7_ – 99.50%) in distillated water (H_2_O). Subsequently, ethylene glycol (C_2_H_6_O_2_ – 99,5%) was added in BiFeO_3_ solution in a 1:2 proportion, and the mixture was stirred at room temperature.

### 4.2 Dip Coating

**FIGURE 4:**
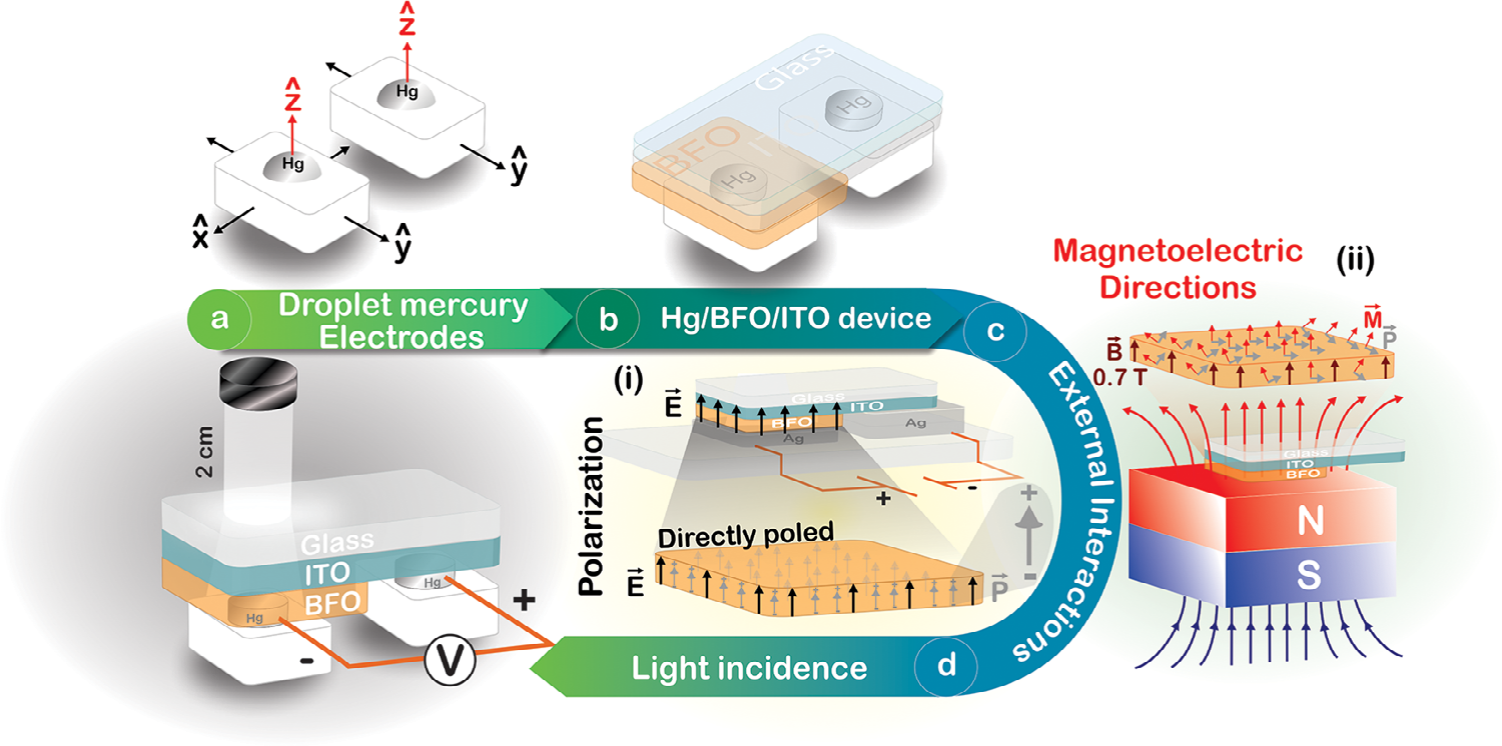
Experimental photovoltaic characterization setup: (a) Hg electrodes, (b) complete setup (glass/ITO/BFO/Hg) of the photovoltaic device, (c) external interactions with magnetic (right) and electric (left) fields, and (d) photovoltaic responses under 200 W light illumination for pristine, poled, and magnetized samples. Insets: Experimental setup: (i) poling process with an applied electric field (E = 0.35 kV/mm) and (ii) magnetization process with an applied magnetic field (H = 0.7 T).

Before dip coating, the ITO‐Glass substrates were cleaned by immersion in acetone under ultrasonic agitation for 10 min and dried in an oven at 50°C air atmosphere for another 10 min. The thin film devices were confectioned by performing 20 cycles of dip coating following these parameters per cycle: constant speed of descent and ascent movements of 10 mm/s, immersion time of 60 s, and drying temperature of 100°C for 15 min. These specific deposition parameters were strictly determined through previous methodological optimization steps to ensure the structural integrity, optimal thickness, and continuous macroscopic formation of the active layer without defects. Finally, the device was heat‐treated at 445°C (heating rate 5 °/min) for 3 h in air atmosphere. The resulting ferroelectric PV device, made with the BFO compound, despite the dip coating cycles, acquired a single active‐layer architecture, i.e., a single active BFO layer of around 450 nm in thickness was deposited on the ITO‐Glass substrate (as shown in Figure 4a), forming a BFO/ITO/Glass device [50].

### 4.3 Photovoltaic Characterizations

Photovoltaic (PV) response measurements of the magnetized, poled, and pristine devices were performed using a custom‐made sample holder connected to an AGILENT 34405A multimeter, as illustrated in Figure 4. The sample holder consists of two electric contacts with individual movements (each with an area of 3.14 mm^2^) made of liquid mercury (Hg), allowing 2D movement for surface scanning (Figure 4a). Subsequently, the PV device (BFO/ITO/glass) was positioned over the electrical Hg contacts (Figure 4b). Electrical point contacts were made on the conductive surface of ITO and the BFO active layer through a z‐shaped movement, completing the electrical circuit. Then, three different settings were used for PV measurements (Figure 4c), i. e., poled (Figure 4—inset (i)), magnetized (Figure 4—inset (ii)), and pristine devices. In this process, a poled BFO layer (gray arrows in Figure 4, inset (i) was obtained after the poling process by applying an external electric DC field of 0.35 kV/mm in z‐direction in steps of 30 min (black arrows in Figure 4, inset (i)). This state is defined in this work as the DP direction. Naturally, if the direction of the electric bias field is reversed, the orientation of electric dipoles and domains should be also reversed, achieving an IP direction.

On the other side, the magnetized BFO layer was obtained by applying an external magnetic bias field (0.7 T) in the z‐direction (brown arrows in inset (ii) of Figure 4). In this arrangement, as expected for BFO, the magnetoelectric coupling must keep the electric dipoles always oriented at 90 degrees relative to magnetic dipoles [43], as illustrated by red (**M**) and gray (**P**) arrows in inset (ii) of Figure 4. However, the typical magnetic dipoles of the BFO compound do not remain fully oriented after removing the magnetic field, resulting in a semi‐oriented electrically and magnetically BFO layer. In fact, a different dipole and domain arrangement from the electric one is observed when DC magnetic fields is applied to the device (magnetic poling), as illustrated in Figure 4—inset (ii). In this arrangement, when magnetic fields are applied perpendicular to the surface device, the magnetic dipoles and domains of the BFO layer should be oriented in the same field direction (perpendicular to the surface of the BFO film). However, once the field is turned off, the interplay between lattice, magnetic, and thermal energies makes it energetically more favorable for the magnetic domains to relax into intermediate orientations, between perpendicular and parallel to the device surface [51]. Consequently, the electric dipoles and domains assume a similar spatially randomized distribution, losing macroscopic polarization. Finally, since charge redistribution follows the dipole and domain rearrangement, regions of high and low electrical potential are expected to form on the device surface, as will be discussed below.

The protocol used for measuring PV responses in the processed devices involved using a Hg‐gas lamp (electrical power of 200 W, broadband emission), positioned 2 cm above the PV device and turned on for 4 min (Figure 4d). The light passed through the glass substrate and ITO layer before reaching the BFO active layer, as shown in Figure 4c. In turn, the photovoltage response was measured using AGILENT 34405A digital multimeter.

### 4.4 Photovoltaic Response of Poled Samples

The PV response was first characterized in a pristine device (unpoled state), as described earlier. After the poling process, the PV characterization was conducted to analyze the points of a typical ferroelectric hysteresis curve, investigating PV responses under saturation polarization, remanence, coercive field, and after reversing the direction of polarization. Thus, the device was directly poled under dc bias electric field (0.35 kV/mm for 30 min), as shown in the Figure 4—inset (i). The inversely poled state was achieved using a similar electric field‐application protocol but in the reverse direction.

### 4.5 Photovoltaic Response of Magnetized Samples

The PV device was magnetized by applying a bias magnetic field (0.7 T for 4 h) obtained from a Nd_2_Fe_14_B permanent magnet (50 × 50 × 200 mm). The magnet is larger than the device, and therefore, the magnetic field was considered to be close to homogeneous across the entire sample. The PV responses of both pristine and directly magnetized BFO devices were measured following the protocol described in Figure 4d. It is important to note that the magnetic saturation values typically reported for BFO, which vary from ∼2 to 3 T for epitaxial films to >9 T for polycrystalline samples, are higher than this field magnitude (0.7 T) [52, 53]. As a result, the magnetic dipoles are not expected to be fully saturated. However, the findings here show that this field intensity, when applied over 4 h period, induces a partial alignment of magnetic domains that can be used to modulate the photovoltaic response via magnetoelectric coupling.

### 4.6 Scanning Electron and Probe Microscopies

AFM, PFM, and KPFM microscopy images were obtained by using a Shimadzu Scanning Probe Microscope (SPM‐9700) equipped with a silica tip covered by a layer of Pt‐Ir, with a spring constant of 0.5‐9.5 N/m and resonance frequency of 4.5‐9.5 kHz. PFM was conducted with electric potentials of ± 1 V. PV responses collected through KPFM images, where the Contact Potential Difference is obtained, were obtained with a halogen lamp (electric power of 50 W, broadband emission), turned on 3 cm over the surface of the BFO thin film. Surface images were obtained by using a scanning electron microscope, Tescan Vega3 XMU in secondary electron (SE) mode, high vacuum, and at 30 kV.

### 4.7 X‐ray Diffraction Analyzes

X‐ray diffraction was performed by using a Shimadzu XRD‐7000 (Cu Kα radiation) diffractometer. The diffraction data results were analyzed by applying the Rietveld refinement method using the FullProf software [42].

## Conflicts of Interest

The authors declare no conflicts of interest.

## Supporting information




**Supporting File**: smtd70625‐sup‐0001‐SuppMat.pdf.

## Data Availability

The data that support the findings of this study are available in the supplementary material of this article and with the author.
