# Supplementary figures and images for: On the Interaction of the Photovoltaic Response With Ferroelectric and Magnetic Domains: Magnetoelectric Control of the Photovoltaic Response in BiFeO3 Thin Films
Source: Small Methods. 2026 Mar 24;10(9):e70625. doi: 10.1002/smtd.70625 (PMC13159401; doi:10.1002/smtd.70625)

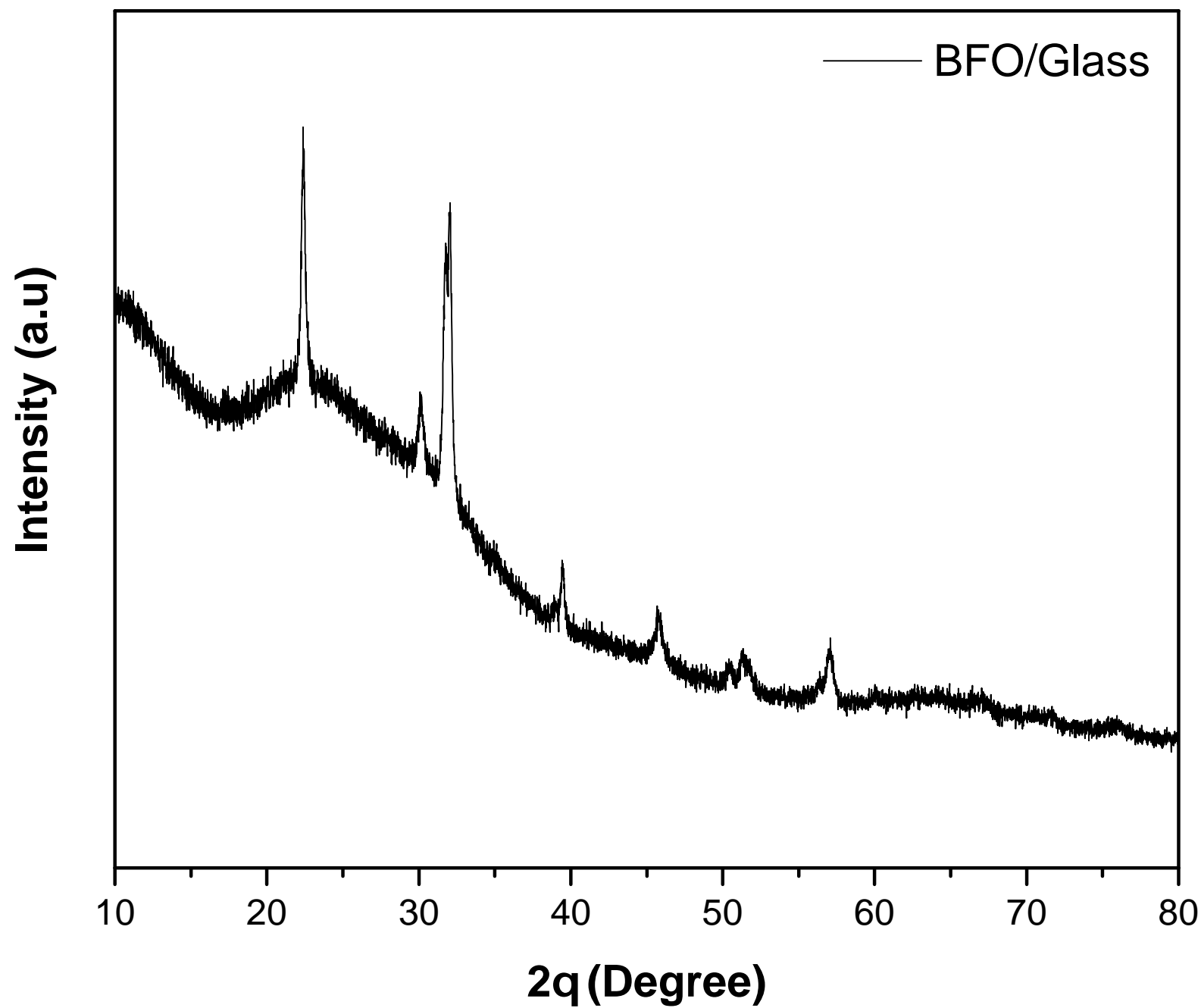

Supplement: Supplementary file 1 — Supporting File: smtd70625‐sup‐0001‐SuppMat.pdf. [file SMTD-10-e70625-s001.pdf]
